# Notch geometry varies with sex and anthropometrics: An analysis on 1043 adults

**DOI:** 10.1002/jeo2.70554

**Published:** 2025-11-28

**Authors:** Cornelius Sebastian Fischer, Max Brenner, Till Ittermann, Julian Constantin Fischer, Robin Bülow, Carsten‐Oliver Schmidt, Lyubomir Haralambiev, Andreas Badke, Tina Histing, Marc‐Daniel Ahrend

**Affiliations:** ^1^ BG Unfallklinik Tübingen, Department of Traumatology and Reconstructive Surgery Eberhard Karls University Tübingen Tübingen Germany; ^2^ Institute for Community Medicine University Medicine Greifswald Greifswald Germany; ^3^ Department of Trauma Surgery Evangelic Hospital Oldenburg Oldenburg Germany; ^4^ Institute of Diagnostic Radiology and Neuroradiology University Medicine Greifswald Greifswald Germany; ^5^ Center for Orthopaedics, Trauma Surgery and Rehabilitation Medicine University Medicine Greifswald Greifswald Germany

**Keywords:** notch geometry, notch depth: notch angle: notch width, notch width index, reference values

## Abstract

**Purpose:**

The geometry of the Notch within the knee joint is highly discussed to influence the overall knee stability and can predict the rupture of the cruciate ligaments. Therefore, associations between anthropometric measurements and notch geometry may help to identify patients at risk for soft tissue knee trauma or inferior surgical outcome. To better describe the normal anatomy of the notch geometry, the primary objective of this study was to examine the notch geometry in a large general population cohort and to define reference values. Furthermore, associations of anthropometric parameters on the notch geometry were examined.

**Methods:**

Notch Depth, Notch Angle, Notch Width and Notch Width Index were measured on bilateral knee magnetic resonance imaging (MRI) of 1043 participants of the Study of Health in Pomerania (SHIP), aged 28–89 years. SHIP drew a sample of the adult general population of Northeastern Germany. Reference values for Notch parameters were assessed by quantile regression models. Associations of sex, age, body height and body weight with the Notch parameters were calculated by linear regression models.

**Results:**

Significantly higher values for men were present for all Notch parameters (*p* = <0.001–0.037) as well as a positive association with age (*p* = 0.001–0.025). Increasing body height was positively associated with Notch Depth and Notch Width (*p* = <0.001), whereas Notch Angle showed an inverse relation to body height (*p* = 0.001). Additionally, Notch Depth showed a significant association with body weight (*p* = 0.009). Based on these associations, adjusted reference values were calculated.

**Conclusion:**

Knee notch geometry is influenced by sex and anthropometric factors. Therefore, individual reference values were provided to enable patient‐specific diagnostics.

**Level of Evidence:**

Level II

**Abbreviations**
ACL: anterior cruciate ligament
BMI: body mass index
ICC: intraclass correlation coefficients

## INTRODUCTION

The knee joint as rotating hinge joint consisting of the tibiofemoral joint and the patellofemoral joint is susceptible for ruptures of the cruciate ligaments due to rotational, extensional and axial trauma. Interindividual differences in bone geometry from both the tibial and the femoral bone are associated with soft tissue injury especially of the anterior cruciate ligament (ACL) [16, 21]. By identifying patients at risk, and by consequently improving modifiable risk factors, a reduction in soft tissue injury might be possible, thereby preventing long‐term damage to the knee joint [8, 12] such as knee osteoarthritis [11]. The geometry of the Notch area within the knee joint as a risk factor is highly discussed to influence the overall knee stability and can predict the rupture of the cruciate ligaments [2, 3, 5, 7]. Therefore, normal values for the Notch parameters and associations between anthropometric measurements and Notch geometry are needed. Several notch parameters are described to quantify the notch geometry: The Notch Angle, the Notch Depth, the Notch Width and the Notch Width Index [22]. The Notch Width in adults ranges from approximately 16–20 mm, with some variation by sex, age and body size [10, 14, 17]. The normal femoral Notch Width Index is considered to be 0.2 [22]–0.27 [18]. Values below this threshold are associated with increased risk of ACL injury [3, 7]. A threshold of < 50° as a critical value for ACL injuries was described by Stein et al. [22] for the Notch Angle. However, the currently published reference values are mostly based on cadaver studies [14], small cohorts [10] or they originate from hospital‐based patients [3, 7, 17, 22].

Therefore, the aim of the present study is to define individualized reference values for notch anatomy. A second aim was to analyse possible associations between inter‐individual differences in Notch geometry and anthropometric attributes such as age, sex, body height and weight. It was hypothesized that femoral notch geometry differs significantly between men and women, and that age as well as anthropometric factors are systematically associated with variations in notch parameters.

## MATERIALS AND METHODS

### Ethics, funding and potential conflicts of interest

The study was approved by the local ethics committee (BB 174/15, 16.12.2015). Each participant gave written informed consent. The Study of Health in Pomerania (SHIP) study is part of the Community Medicine Research Net of the University of Greifswald, Germany, which was funded by the Federal Ministry of Education and Research (grant No. 03ZIK012), the Ministry of Cultural Affairs, as well as the Social Ministry of the Federal State of Mecklenburg‐West Pomerania. Magnetic resonance (MR) imaging was supported by the Federal State of Mecklenburg‐Vorpommern, the Federal Ministry of Education and Research and a joint grant from Siemens Healthcare, Erlangen, Germany. Each author certifies that no competing interests exist.

### Design and sample

The data were assessed within the SHIP. SHIP was designed as a cross‐sectional study and is an ongoing population‐based project. It consists of two independent cohorts, SHIP‐START and SHIP‐TREND. To ensure a general population cohort, participants were randomly recruited from official resident registry office files and stratified by gender, age and city of residence. The samples for both cohorts were drawn from a defined region in Northeastern Germany. The sample for SHIP‐TREND was drawn in 2008. In the baseline assessment of SHIP‐TREND‐0, 4420 individuals were examined (response 50.1%). The final follow‐up of SHIP‐TREND‐1, considered in the present study, was conducted between 2016 and 2019 with a response rate of 2507 participants (56.7%) [24].

In SHIP‐TREND‐1, all participants had the option to receive a magnetic resonance imaging (MRI) of both knees in addition to the standard whole‐body MRI. MRI dropouts were caused, for instance, by claustrophobia, metal implants or personal reasons. For the present study, all participants from SHIP‐TREND‐1 with a completed knee MRI protocol were included.

### MRI protocol and angular measurements

The MRI was performed with a 1.5‐T MR scanner (Magnetom Avanto; Siemens Medical Systems). All MRI examinations were performed in a supine position in a standardized manner. The MRI was acquired using a three‐dimensional (3D) proton density fat‐saturated space sequence with a repetition time (TR) of 1200 msec; echo time (TE) of 30 msec; 120° flip angle; field of view 180 × 180 mm; matrix 256 × 100; and bandwidth, 698 Hz/pixel with a resulting voxel size of 0.7 × 0.7 × 0.7 mm. The sequence was performed in a neutral position lying on the back.

All measurements on MRI were performed by one trained observer, blinded to all information about the participants, using OsiriX version 13.0.2 (PIXMEO). For reliability assessment of the knee geometric parameters, 26 cases were measured twice for intraobserver variability (M. K.) and a third time (C. F.) for interobserver variability.

The Notch and condylar measurements were performed on the axial view as described in previous literature [4, 9, 22].

**Figure 1:**
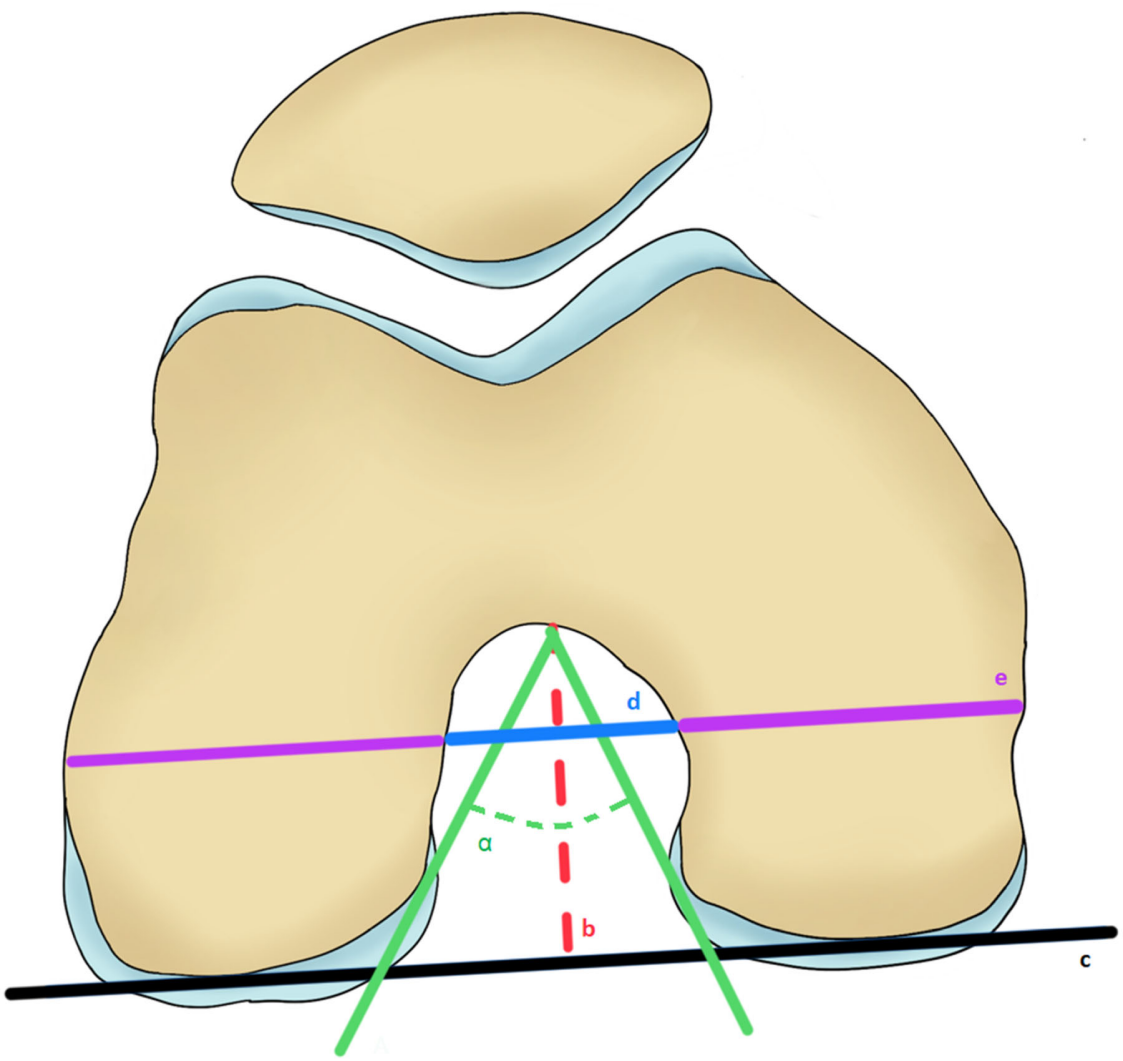
Measurements of the femoral Notch. The green lines form the Notch Angle α. The Notch Depth (b) is perpendicular to the surfaces of the medial and the lateral femur condyle (c). Notch Width (d) and condylar width (e) are parallel to c at 2/3 of b.

The Notch Angle was defined as the angle between the most anterior point of the Notch and the medial as well as the lateral aspect of the inner femur condyles. The Notch Depth is the perpendicular distance from the most anterior point of the Notch to a line connecting the articular surfaces of the femoral condyles. The Notch Width and the femur width are measured parallel to the connecting line of the articular surfaces of the femoral condyles at 2/3 of the Notch Depth. Notch Width Index is calculated by the ratio between the Notch Width and the width of the femur (Figure 1).

### Anthropometric measurements

Body weight and height were measured to the nearest 0.1 kg and 0.5 cm, respectively, using calibrated weighing scales and stadiometers with the participant wearing light clothing without shoes. Body mass index (BMI) was calculated as weight (kg)/height^2^ (m^2^).

### Statistics

Intraobserver variability and interobserver variability were evaluated by intraclass correlation coefficients (ICC). Standard descriptive statistics such as Mean ± SD (min–max) and percentiles were used to describe the study cohort. All reported *p* values were two‐tailed; *p* values < 0.05 were considered statistically significant. The correlation of notch geometry parameters between both sides (left and right) were analysed using Spearman's correlation coefficient. Associations of demographic and anamnestic data, including possible interactions between the parameters, with notch geometry parameters were determined by linear regression analyses. Fractional polynomials for potential nonlinear associations were tested between age and the angles. Interactions between age and sex were tested, and *p* < 0.1 was considered statistically significant for these analyses. Stratified by sex, age‐specific upper and lower reference limits were calculated by quantile regressions for the 2.5th and 97.5th percentiles. Reference values were calculated by the 95% reference interval (mean ± 1.96 SD). Normality of the parameters were checked visually, and all notch parameter were approximately normally distributed (Supporting Information). All statistical analyses were performed using Stata 18.5 (Stata Corp.).

## RESULTS

### Sample description

**Figure 2:**
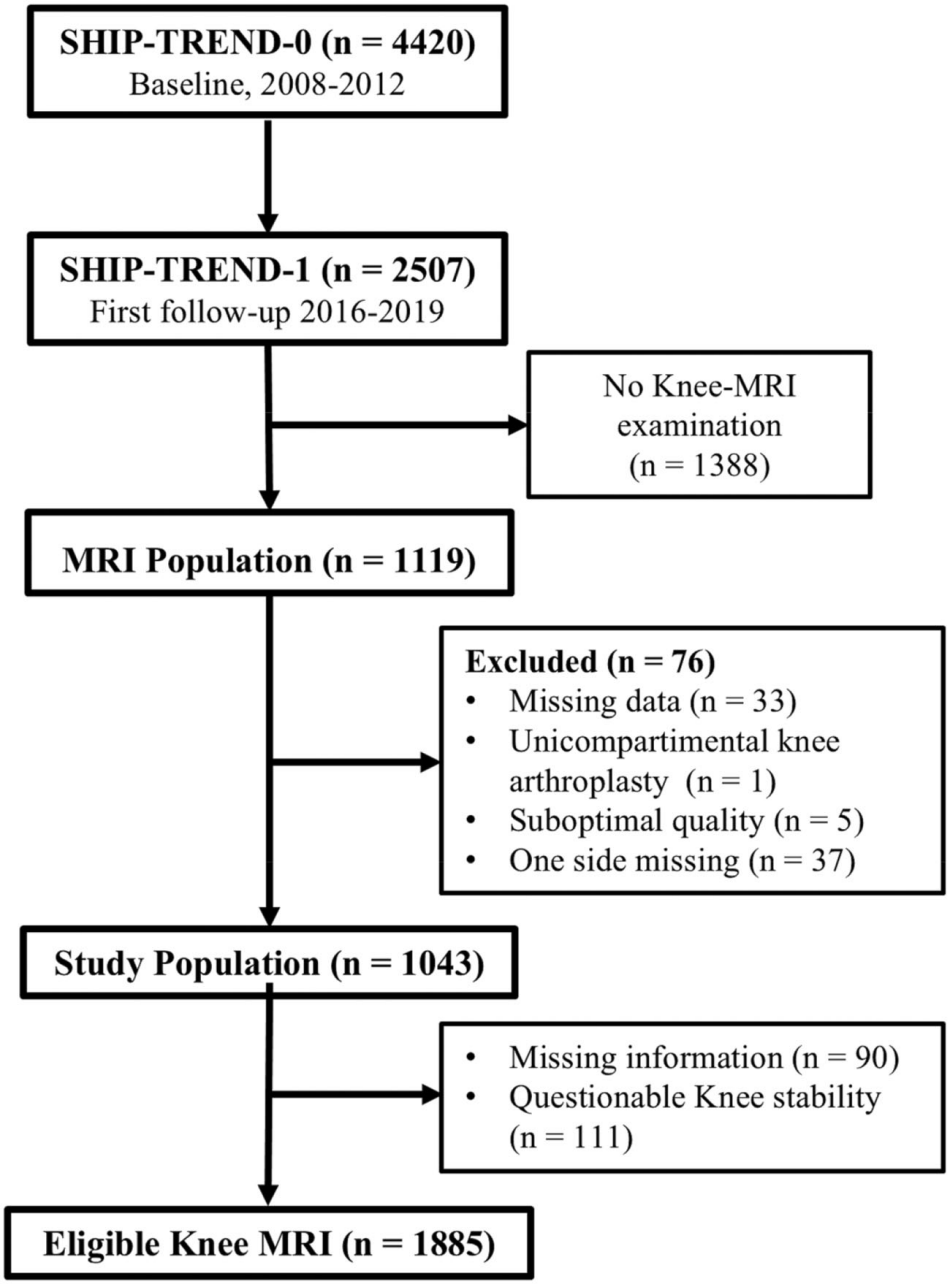
The flow diagram illustrates the selection of the study population. MRI, magnetic resonance imaging.

Overall, 1119 individuals participated in the knee MRI examination. Thirty‐nine participants had to be excluded because of missing data (33), unicompartimental knee arthroplasty (1) or suboptimal MRI quality (5). In total, 1043 participants were eligible. Additionally, 90 knee joints were excluded due to missing data on stability. In 111 knee joints, questionable stability was detected by the drawer test. Therefore, those knee joints were excluded as well (Figure 2).

The mean age of the 1043 participants (50% female) was 56.2 (28–89) years. The mean values of body weight, body height and BMI were higher in males than in females. The sample description is shown in Table 1. A comparison between the baseline (SHIP‐TREND‐0) and the present sample is shown in Table 2.

**Table 1:** Data for the study population.

	Males (*n* = 522)			Females (*n *= 521)		
	Mean	SD	Min‐max	Mean	SD	Min‐max
Age (years)	55.6	13.1	28–87	56.7	12.0	29–89
Body height (cm)	177.2	7.0	158–195	164.1	6.4	145–185
Body weight (kg)	87.1	12.7	51.3–124.3	71.9	12.1	44.1–112.9
Body mass index (kg/m^2^)	27.7	3.4	17.4–37.9	26.7	4.3	18.2–41.2
Notch Depth (mm)	31.1	2.1	23.4–37.3	28.0	1.8	22.9–33.4
Notch Angle (degree)	56.8	7.3	35.1–81.9	57.0	6.5	38.9–78.2
Notch Width (mm)	21.7	2.1	15.5–28.5	19.3	1.8	14.4–24.4
Notch Width Index	0.28	0.02	0.21–0.36	0.29	0.03	0.22–0.37

*Note*: Continuous data are given in means and standard deviations; categorical data in absolute numbers.

Abbreviation: SD, standard deviation

**Table 2:** Data for the study population based on the SHIP‐TREND‐0 cohort.

	Excluded (*n* = 3377)	Included (*n* = 1043)
Age (years)	52.9 (16.1)	48.9 (12.6)
Sex		
Male	1623 (48.1%)	522 (50.0%)
Female	1754 (51.9%)	521 (50.0%)
Body height (cm)	169.4 (9.4)	171.2 (9.3)
Body weight (kg)	82.0 (17.6)	78.9 (14.1)

### MRI readings

Good variability for the assessment of the knee geometric parameters was achieved with ICCs ranging between 0.841 and 0.978 for intraobserver variability, and ICCs between 0.851 and 0.987 for interrater variability. The mean difference and the standard error of the variability assassment are presented in the supplements.

**Figure 3:**
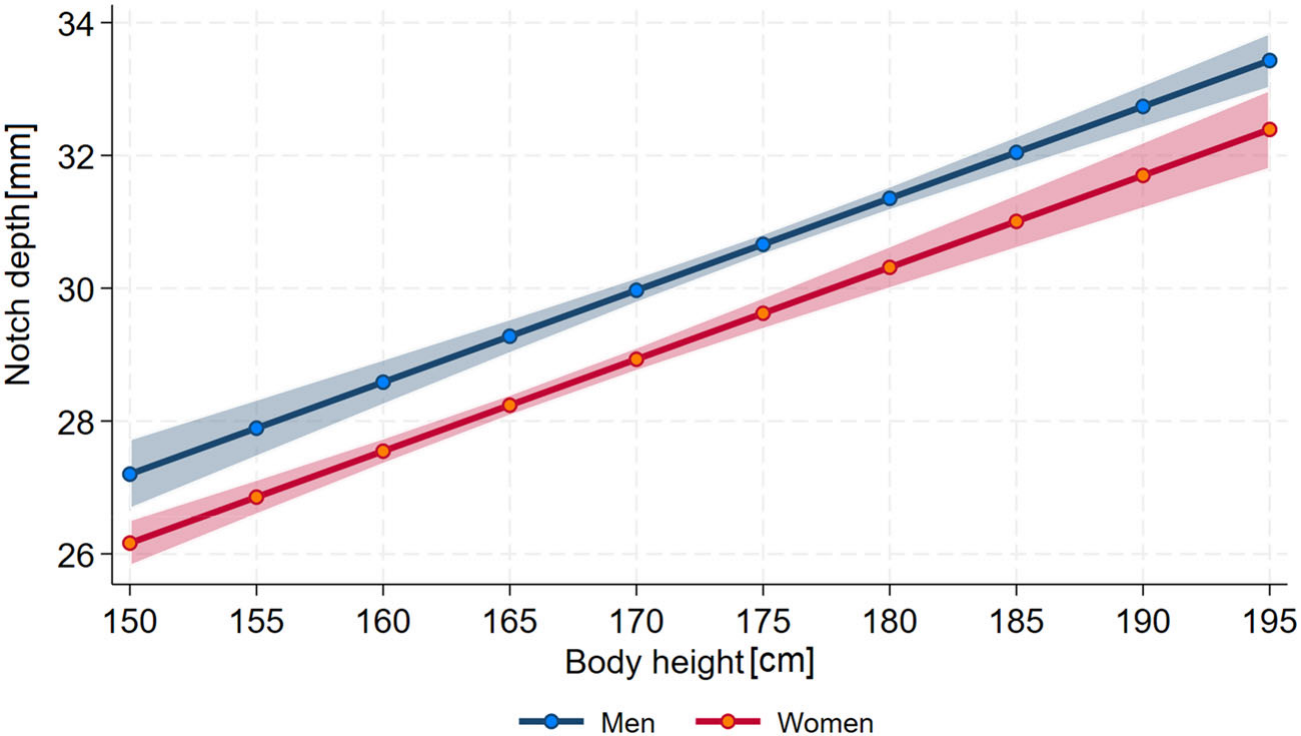
Association between Notch Depth and body height for both genders.

The mean Notch Depth for all 2086 knees (both sides) was 29.6 mm with a range of 23.1 mm–37.6 mm. The Notch Angle showed a range of 35.3°–85.4° with a mean of 56.8°. The mean Notch Width was 20.8 mm and ranged from 14.5 mm up to 29.5 mm while the Notch Width Index had a mean of 0.29 (range 0.21–0.37). Details on the notch geometry according to sex are shown in Table 1. All parameters showed a good correlation between the left and right knee (Notch Depth *r* = 0.8995; Notch Angle *r* = 0.7994; Notch Width *r* = 0.8010; Notch Width Index *r* = 0.6606). The Notch Depth showed significantly higher values for men (*p* = <0.001). Body height (*p* = < 0.001) and age (*p *= 0.019) were positively associated with Notch Depth for men and women (Figure 3).

**Figure 4:**
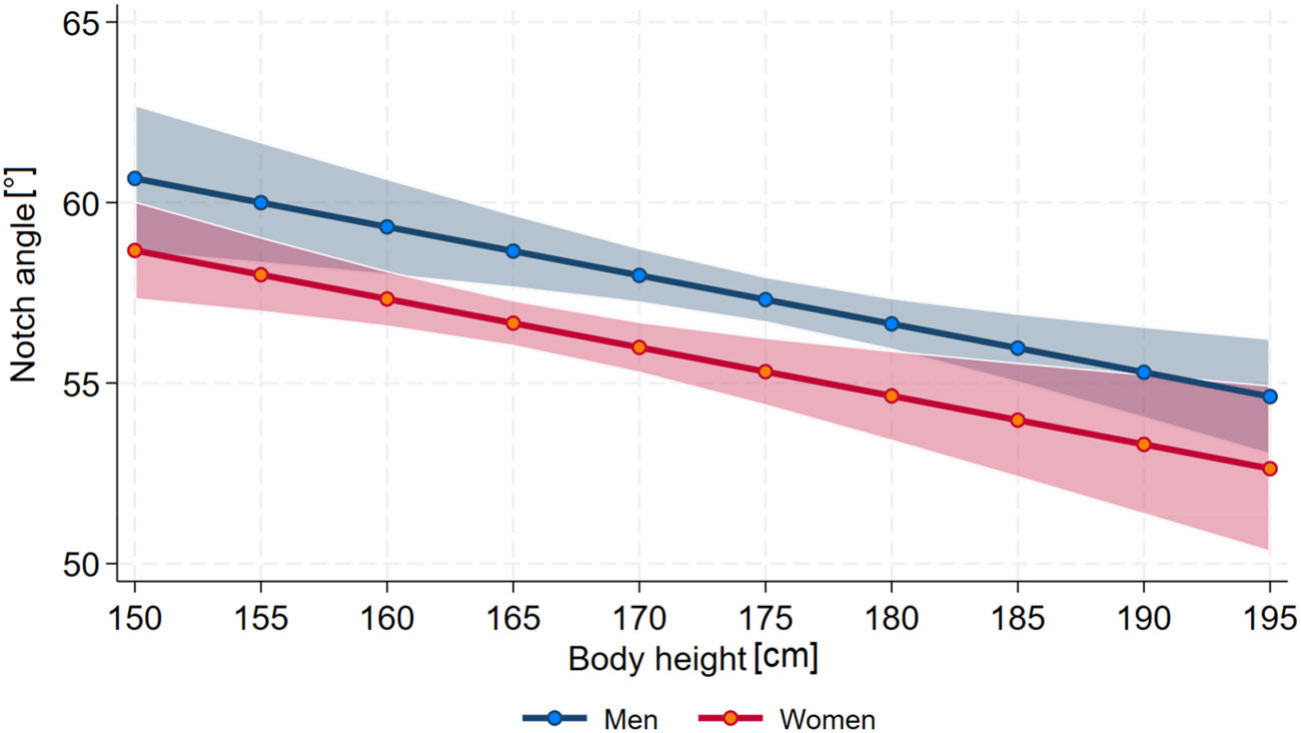
Association between Notch Angle and body height for both genders.

The Notch Angle was positively associated with age (*p* = 0.025) and inversely associated with body height (*p* = 0.001, Figure 4).

**Figure 5:**
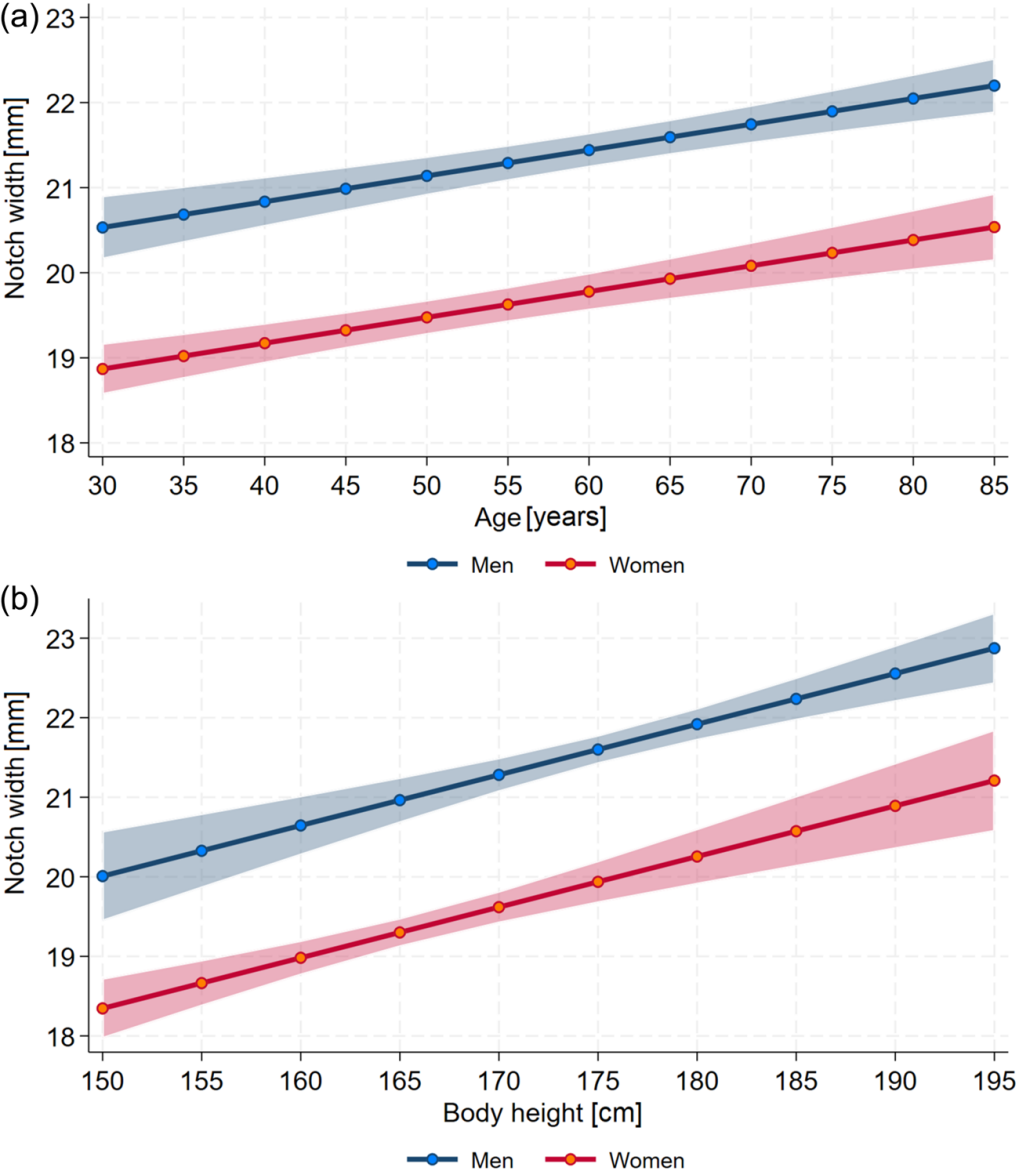
Association between Notch Width and (a) age and (b) body height for both genders.

Significantly positive associations were present between age (*p* = 0.001) as well as body height (*p* = < 0.001, Figure 5) with Notch Width, while men showed higher values than women (*p* = < 0.001).

Age was positively associated with the Notch Width Index (*p *= 0.021) with higher values for men (*p* = 0.037).

Body weight was not associated with Notch Angle (*p* = 0.155), Notch Width (p = 0.766) and Notch Width Index (*p* = 0.194), whereas a significant association was present with Notch Depth (*p* = 0.009).

The assessed reference values for the Notch parameters are shown in Table 3. Due to the discovered associations, adjusted reference values for women and men were assessed and can be calculated by the formulas as presented in Table 4.

**Table 3:** Reference values for the Notch parameters, defined on stable knee joints (*n* = 1885).

Parameter	Mean	SD	95% reference interval
Notch Depth (mm)	29.57	2.59	24.49–34.65
Notch Angle (degree)	56.86	7.18	42.79–70.93
Notch Width (mm)	20.53	2.41	15.81–25.25
Notch Width Index	0.28	0.03	0.22–0.34

Abbreviation: SD, standard deviation.

**Table 4:** Reference values for Notch parameters adjusted for gender, age (years), body height (cm) and body weight [kg].

	2.5th percentile	97.5th percentile
Notch Depth (mm)	‐3.52138 + 0.032185*age + 0.2217349 (W) + 0.1477391*BH + 0.0282841*BW	2.37128 + 0.0228219 *age ‐ 1.368313 (W) + 0.1767417*BH + 0.0008562*BW
Notch Angle (degree)	66.40245 ‐ 0.0204141*age ‐ 0.039623 (W) ‐ 0.1278463*BH ‐ 0.0020971*BW	117.1652 ‐ 0.0685466 *age ‐ 4.382653 (W) ‐ 0.2177004*BH ‐ 0.0397314*BW
Notch Width (mm)	6.538149 + 0.0197549*age ‐ 1.365608 (W) + 0.0603577*BH + 0.0094211*BW	12.59676 + 0.0300354*age ‐ 2.075202 (W) + 0.0625782*BH + 0.0059662*BW
Notch Width Index	0.2399777 + 0.0001659*age ‐ 0.0018134 (W) ‐ 0.0000127*BH – 0.0001975 *BW	0.3543271 + 0.0000257*age + 0.0059253 (W) ‐ 0.000082*BH ‐ 0.000055*BW

Abbreviations: BH, body height in cm; BW, body weight in kg; (W), women.

## DISCUSSION

The present study detected altered Notch Geometry according to sex and anthropometrics. Significantly higher values for Notch Depth and Notch Width in men compared with women were present. Additionally, positive associations between body height and Notch Depth and Width as well as an inverse association between body height and Notch Angle was observed. Furthermore, body weight was positively associated with Notch Depth.

Patients with altered Notch geometry are vulnerable for knee pathologies; therefore, information on reference values of the Notch and associated factors might be valuable for the identification of patients at risk. The present study examined the associations between age, sex, body height and weight with intercondylar Notch geometry in a large cohort of the population‐based SHIP study.

In consistency with previous research, the present study shows significantly greater Notch Depth in men compared with women [8] as well as wider Notch Width [8, 21]. Contradicting these findings, Carlton et al. [10] did not observe significant differences in gender for femoral Notch Width in an analysis of 91 knees from 48 patients. A significant difference was found considering weight. Heavier patients had a wider femoral Notch Width compared with lighter patients. No significant association was found between height and Notch Width. These opposing findings could be due to the relatively small study group and a young age of the participants ranging only from 20 to 34 years [10]. Furthermore, participants of the present study are not patients with knee disorders, but part of a population‐based cohort.

Interestingly, the notch parameters increased with age in the present cohort. A possible explanation for this observation could be age‐related remodelling or cortical thinning due to degenerative changes in the bone. However, the clinical relevance of this result might be inferior.

Additionally, positive associations between age and body height with the Notch Angle were observed, along with greater notch angle values for men compared with women. There were no associations between body weight and the notch angle. Balgovind et al. described similar associations in an Indian population of 50 participants with a mean Notch Angle of 54.16° for men and 51.03° for women [5].

While previous studies have identified a critical notch angle of 50° [4, 9, 22], the present study demonstrates a broader range, from 42.79° to 70.93°, with a mean of 56.86°. This discrepancy may be attributed to differences in study populations. Stein et al. [22] analysed 160 participants from the Osteoarthritis Initiative Study, whereas Anderson et al. [4] examined 48 individuals aged 23–26 years, two‐thirds of whom had known ACL injuries. Furthermore, Cha et al. reported a mean notch angle of 50.3° in 105 patients, 47 of whom had undergone arthroscopic notchplasty [9]. In contrast, the present findings are based on a population‐based cohort with a broader age distribution without selection based on specific pathologies. As such, the results may reflect the variability present in the general population more accurately. Alternating personal anthropometric characteristics play an increasing role in modern, individualized medicine. Therefore, patients’ individual anatomic characteristics should be considered before and after knee surgery. Huang et al. showed a smaller Notch Angle in patients with ACL tear compared with those without, linking Notch geometry to ACL injury [19]. Additional studies associated ACL rupture with a narrow Notch as well as decreased Notch Width Index ( < 0.270) [3, 7]. Research by Fahim et al. on a sample of 60 participants observed a higher incidence of ACL injuries in patients with narrower Notch (Notch width 19.9 mm) compared with those with wider Notch [15]. Furthermore, they determined that a lower Notch Width index (0.27) was associated with ACL tear, while males had lower Notch Width index than females. As a cut‐off value to predict ACL tears, a Notch Width index of 0.29 was determined [15]. For the present population, a reference value for Notch Width index between 0.22–0.34 was calculated, expanding the physiological values stated by Fahim et al. [15]. Due to the large number of measurements, it was possible to define reference values according to gender, age, body height and weight, which is a new finding and highlights the importance of the current study.

The geometry of the Notch does not only play an important role in the development and prediction of pathological states of the knee joint, but it is also important in the operative treatment of ACL injuries, because a narrow Notch Width could lead to poor outcomes of ACL grafts and graft rupture [1, 20, 23]. Levins et al. showed, for example, for every 1 mm increase in Notch Width, a decreasing risk by 28% for ACL graft rupture in females [20]. These findings are in line with previous studies in both adults [3, 8] as well as adolescents [13]. For example, Cay et al. observed lower Notch Width in patients with ACL degeneration (18.2 + –3.1 mm), partial ACL tear (17.3 + −2.5 mm) and complete tear (17.1 + −2.6 mm) compared with a control group with healthy ACL (19.1 + −2.9 mm) [8]. A case report consisting of four patients with ACL‐graft by Akamatsu et al. observed in second‐look arthroscopy either cyclops‐like lesions or partial tears of the ACL‐graft at the contact point between ACL graft and intercondylar Notch, requiring subsequent notchplasty in all cases [1]. Therefore, modifying the Notch geometry while ACL reconstruction should be considered. Other authors did not find any associations between small intercondylar Notch dimensions and ACL graft tear; consequently, notchplasty is not recommended during ACL reconstruction [25].

One potential limitation of the study may be that MR imaging is inferior to computed tomography (CT) for detailed assessment of bony structures. Nevertheless, most studies on notch geometry use MRI [5, 6, 8, 10]. Furthermore, MRI is the standard diagnostic tool for ACL ruptures. Therefore, providing reference values based on MRI appears reasonable. Another limitation is the generalizability of the results to other populations, as only European individuals from a specific geographic region were investigated. However, no ethnic differences have been reported in the current literature. Furthermore, the representativeness of the present population from rural Pomerania may be limited due to participant loss during follow‐up. However, through the randomized selection mechanism and the stratification, with only minor differences compared with the baseline cohort, the presented results can still be considered reliable for European white populations. Additionally, severe pathology is often treated by surgery with remnants effortlessly detectable in MR imaging. Another limitation is that only adults were examined, so any transferability to children or adolescents should be made with caution. In contradiction, other studies focused mainly on specific study populations like athletes [6], adolescents [2] or consisted of small study samples [5, 6]. Therefore, they cannot be considered representative for a general population. Consequently, the defined threshold values are of high relevance for clinical routine since they can help physicians assessing knee pathologies adjusted to the patient‐specific attributes. A practical way to facilitate clinical use of the formulas would be their implementation in an Excel spreadsheet. Thus, the resulting therapy can be planned more accurately.

## CONCLUSION

This study examined the Notch geometry in a large general population, defining reference values of the Notch geometry regarding age, sex, body height and weight. Significantly higher values for all Notch parameters in men compared with women were revealed and associations between increasing body height and greater Notch Depth and Notch Width as well as smaller Notch Angle were found. In modern individualized medicine, altering personal and anthropometric characteristics play an increasing role. The adjusted formulas provided by this study take these patient characteristics in consideration. The individual reference values offer the possibility of patient‐specific diagnostics and therapy.

## AUTHOR CONTRIBUTIONS

**Cornelius Sebastian Fischer**: Conceptualization; data curation; validation; investigation; methodology; visualization; project administration; supervision; writing—original draft. **Max Brenner**: Conceptualization; investigation; formal analysis; methodology; software, writing— review and editing. **Till Ittermann**: Validation; software; formal analysis; writing—review and editing. **Julian Constantin Fischer**: Visualization; writing—original draft. **Robin Bülow**: Data curation; writing—review and editing. **Carsten‐Oliver Schmidt**: Data curation; methodology; writing—review and editing. **Tina Histing**: Writing—review and editing. **Lyubomir Haralambiev**: Writing—review and editing. **Andreas Badke**: Writing—review and editing. **Marc‐Daniel Ahrend**: Conceptualization; methodology; project administration; supervision; writing—review and editing.

## CONFLICT OF INTEREST STATEMENT

The authors declare no conflict of interest.

## ETHICS STATEMENT

This study was approved by the Ethics Board of the University of Greifswald (Approval Number: BB 174/15, 16.12.2015).

## Supporting information

Supporting information.

## Data Availability

The data that support the findings of this study are publicly available for sicentific purposes, and those interested can apply for data usage at the Institute for Community Medicine, University Medicine Greifswald, Greifswald, Germany.
