# Supplementary material for: Notch geometry varies with sex and anthropometrics: An analysis on 1043 adults
Source: J Exp Orthop. 2025 Nov 28;12(4):e70554. doi: 10.1002/jeo2.70554 (PMC12661209; doi:10.1002/jeo2.70554)

Supplemental material

**Mean difference (standard error) for intra- and inter-reader measurements**

|  | **Intra-reader** | **Inter-reader** | **Intra-reader** | **Inter-reader** |
| --- | --- | --- | --- | --- |
|  | **Right knee** | | **Left knee** | |
| **Notch Depth [mm]** | -0.01 (0.85) | 0.29 (0.80) | 0.22 (1.00) | 0.45 (1.02) |
| **Notch Angle [degree]** | 0.46 (3.97) | -1.07 (3.77) | 0.73 (4.43) | -0.92 (4.18) |
| **Notch Width [mm]** | 0.35 (0.89) | -0.26 (0.89) | 0.52 (0.94) | -0.34 (0.89) |
| **Notch Width Index** | 0.01 (0.02) | -0.01 (0.02) | 0.01 (0.02) | -0.01 (0.02) |

Based on linear regression models with reader (measurement 2 vs measurement 1 [intra] or measurement 1 vs gold standard [inter]) as independent variable. Results are reported as β and standard error.

**Histograms of the Notch measurements** **to asses normality**


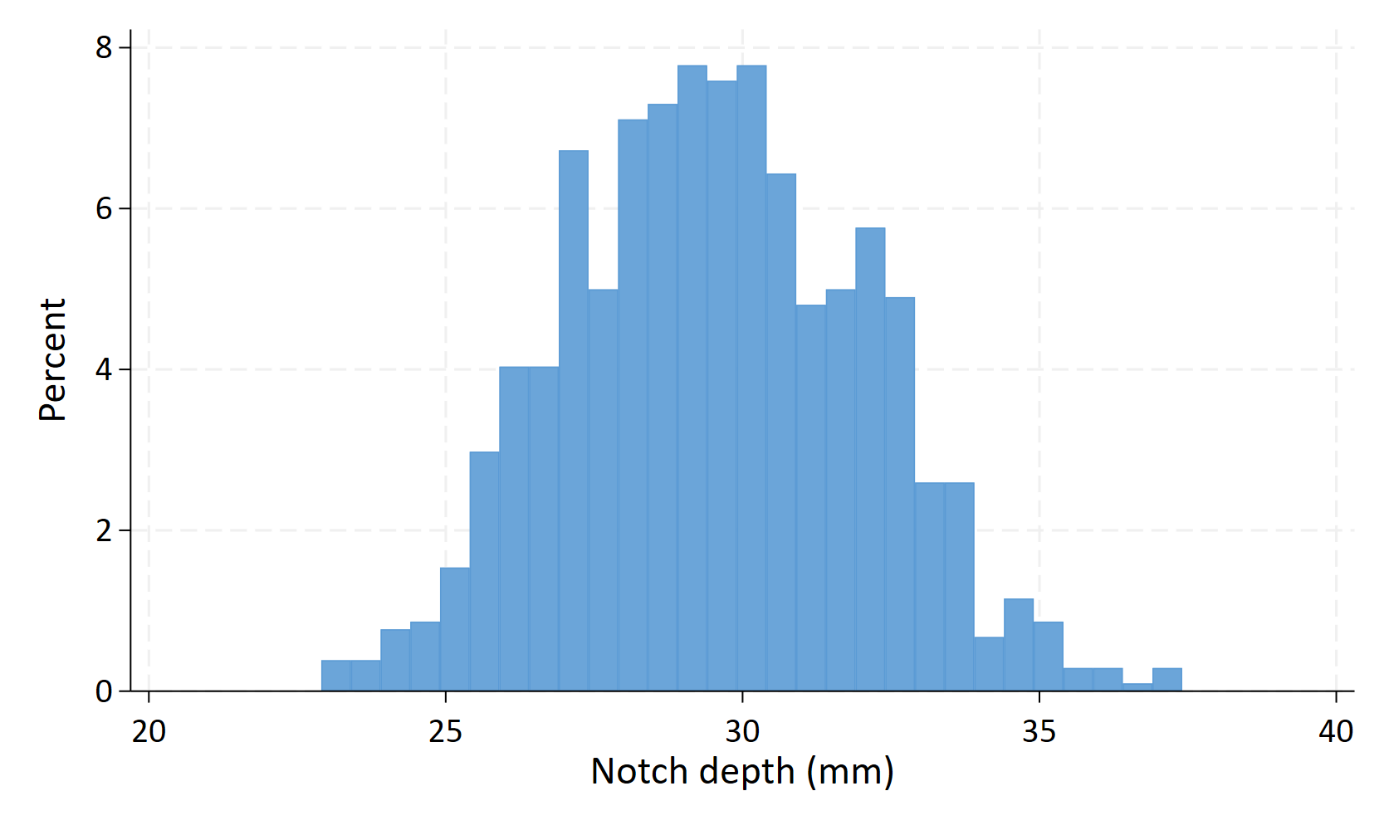


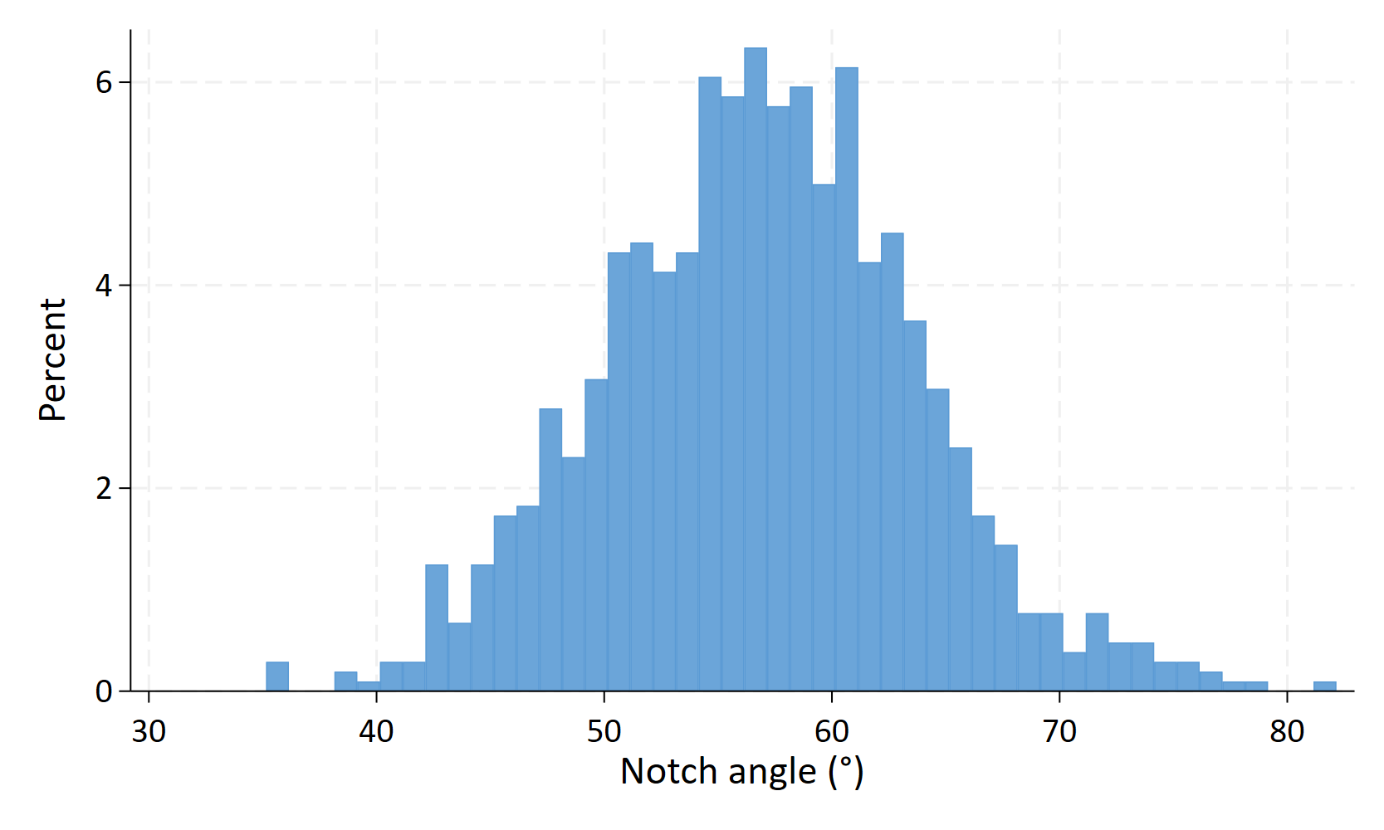


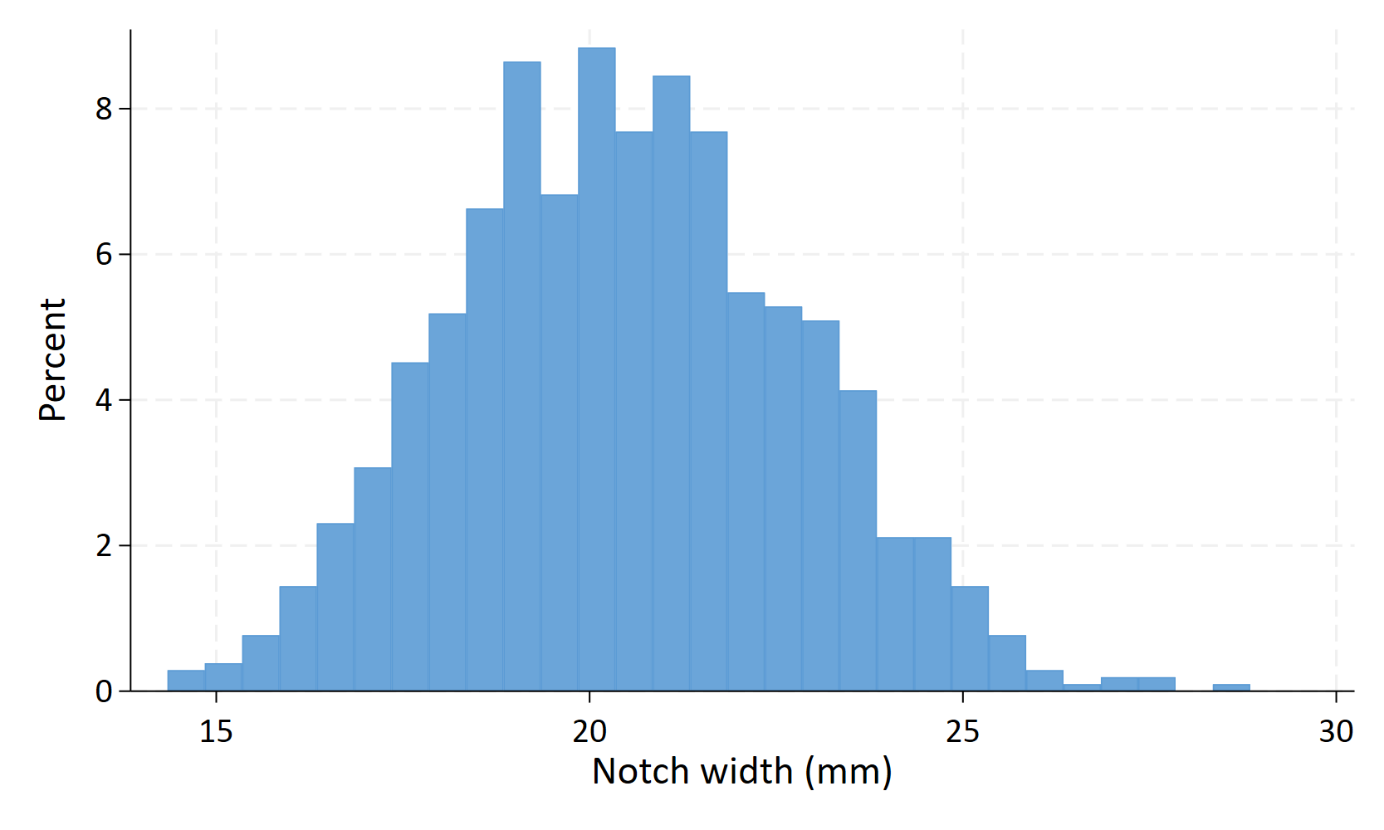


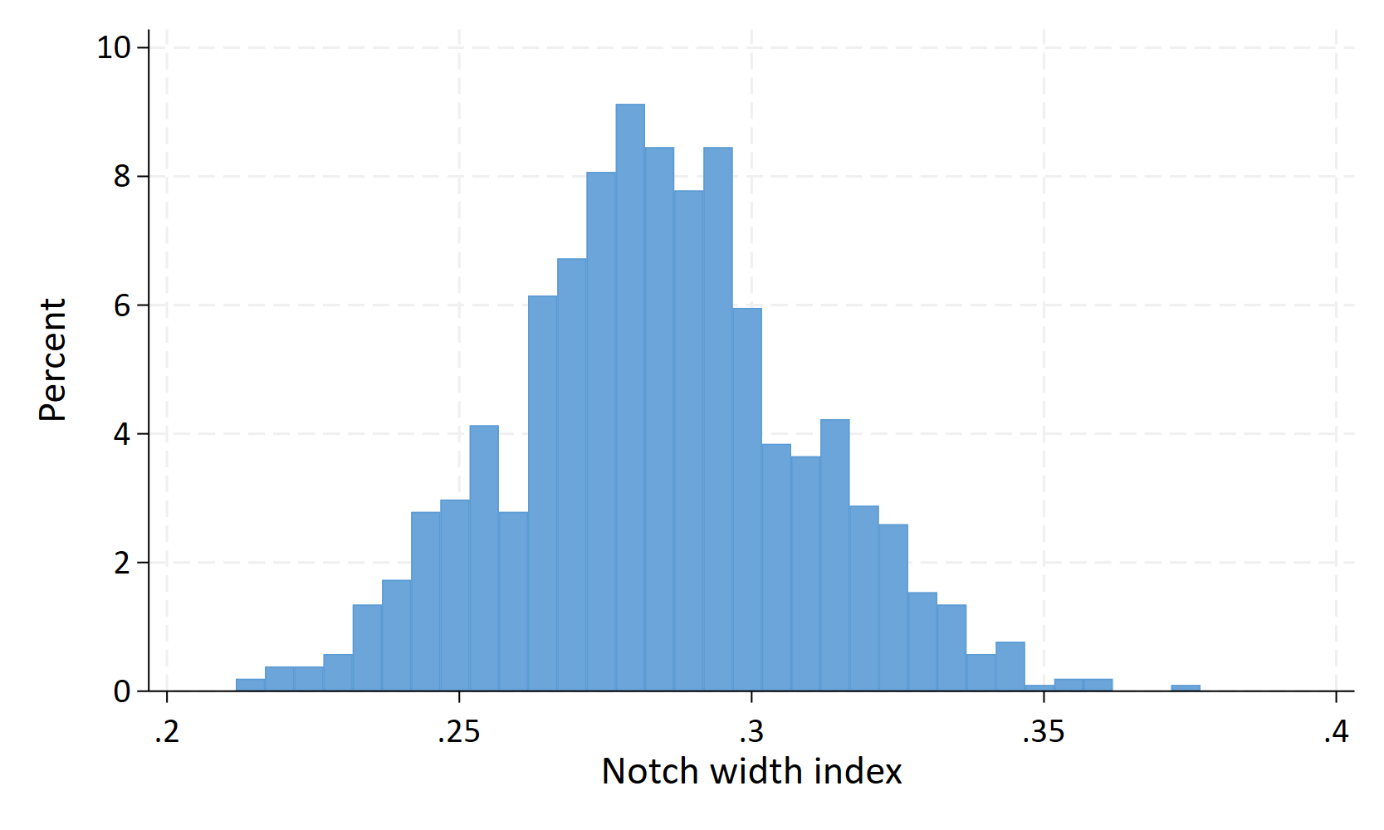

Supplement: Supplementary file 1 — Supporting information. [file JEO2-12-e70554-s001.docx]
